# Microbial Composition, Disease Trajectory and Genetic Background in a Slow Onset Model of Frontotemporal Lobar Degeneration

**DOI:** 10.3390/biom15050636

**Published:** 2025-04-29

**Authors:** Nathalie Daude, Ivana Machado, Luis Arce, Jing Yang, David Westaway

**Affiliations:** Centre for Prions and Protein Folding Diseases, University of Alberta, Edmonton, AB T6G 8M8, Canada; ndaude16@gmail.com (N.D.); jyang6@ualberta.ca (J.Y.)

**Keywords:** tauopathy, protein misfolding, microbiome, transgenic mouse, frontotemporal dementia, neurodegeneration, neuroinflammation, inbred strain

## Abstract

Slow-onset neurodegenerative disease in a low-expresser 2N4R P301L transgenic (Tg) mouse model is marked by neuroinflammation and by differing patterns of CNS deposition and accumulation of tau conformers, with such heterogeneities present even within inbred backgrounds. Gut microbial genotypes were notably divergent within C57BL6/Tac or 129SvEv/Tac congenic (Cg) sublines of TgTau^P301L^ mice, and these sublines differed when challenged with antibiotic treatment and fecal microbial transplantation. Whereas aged, transplanted Cg 129SvEv/Tac TgTau^P301L^ mice had neuroanatomical deposition of tau resembling controls, transplanted Cg C57BL6/Tac TgTau^P301L^ mice had different proportions of rostral versus caudal tau accumulation (*p* = 0.0001). These data indicate the potential for environmental influences on tau neuropathology in this model. Furthermore, Cg C57BL6/Tac TgTau^P301L^ cohorts differed from 129SvEv/Tac counterparts by showing 28% versus 9% net intercurrent loss (*p* = 0.0027). While the origin of this phenomenon is not established, it offers a parallel to differing patterns of frailty observed in C57BL6 versus 129 SvEv Tg mice expressing the 695 amino acid isoform of human amyloid precursor protein. We infer that generalized responses to protein aggregation might account for similar reductions in viability even when expressing different human proteins in the same inbred strain background.

## 1. Introduction

Alzheimer’s disease and related disorders (ADRD) place enormous societal, financial, logistic, and emotional burdens upon aging populations [1,2]. Within the spectrum of ADRD, frontotemporal dementia (FTD) can be subdivided into different types, including those manifesting with linguistic or behavioral alterations [3,4]. Degeneration of frontal areas revealed by in vivo imaging or by postmortem histological analyses is prominent [5], with frontotemporal lobar degeneration (FTLD) being a particular version of FTD defined from the neuropathological status of brain material. Via a breakthrough observation, certain cases of FTD with dominant Mendelian inheritance were traced to mutations in the tau gene (*MAPT*) [6]; this observation aligns with florid deposition seen in FTLD-MAPT cases by immunohistochemistry with phospho tau antibodies [7,8]. While some disease-causing *MAPT* mutations affect pre-mRNA splicing, others correspond to missense changes that map to a protease-resistant microtubule-binding repeat region of the tau protein, and knowledge of these mutations (reviewed in [9]) has been leveraged to make a variety of transgenic (Tg) rodent models of FTLD. Notably, such models—even with ostensibly similar specifications such as expression from a constitutive neuronal promoter and the inclusion of P301L and P301S missense mutations—have different attributes [10,11,12]. Recently, we have been profiling a mouse Tg line with extremely stable, low-level expression of the 2N4R spliced from tau with a P301L mutation. This Tg model, henceforward called TgTau^P301L^ mice) has tau expression in the brain 1.7× above endogenous levels [13,14] and develops florid tau pathology in neurons, astrocytes, and oligodendrocytes. Functional impairments to motor function have not been noted in this model, in contrast to other tau transgenics [15]. Prior analyses noted that the timing of onset of these neuropathological changes differed in two breeding colonies (faster in a Japanese colony, slower in a Canadian colony [14]). While this effect suggested environmental inputs on disease-related endpoints, the FVB/N × 129 F1 mixed genetic background used for Tg production [14] also suggested a role for segregation of chromosomal modifier loci. However, the production of congenic derivatives of the founder line did not eliminate variations in neuropathology [13]. Similarly, heterogeneity was seen in congenic TgTau^P301L^ mice when profiled for the chemical denaturation behavior of sarkosyl-insoluble, disease-associated tau conformers or using protease digestion and mass spectrometric analysis [16]. The observation of collections (ensembles) of disease-associated tau conformers in brain material from an extended FTLD-MAPT-P301L pedigree argues that the heterogeneity of tau conformers is not an idiosyncrasy of the TgTau^P301L^ model; rather, complex denaturation profiles were sometimes similar between FTLD-MAPT-P301L patients and TgTau^P301L^ mice [16]. There is an open question about the cell biological heterogeneity of tau deposits in human conditions [17] and, in other contexts, heterogeneity has been laid at the door of protein conformation; in neurodegenerative disease caused by misfolding of the cellular prion protein PrP^C^, ensembles of different conformers have been equated with “clouds” of self-templating protein strains [18].

Given the growing interest in the gut–CNS signaling in the pathogenesis of human neurodegenerative diseases [19,20] and AD [21,22,23] and an open question about the origin of heterogeneity within TgTau^P301L^ mice, we considered whether the gut microbiome might be of relevance. Specifically, we genotyped gut microbiota in two congenic derivatives of TgTau^P301L^ mice and then asked whether changes in pathology could be influenced by changes in microbiota, a finding that might have practical implications. To this end, we carried out microbial transplantation studies using fecal material from aged TgTau^P301L^ mice that had different neuroanatomical patterns of AT8-positive tau deposition. We then scored transplanted mice in later life to assess whether pathological outcomes are matched to the type of microbial transplant input.

## 2. Material and Methods

### 2.1. Transgenic Mice

TgTau (P301L) 23027 mice (for brevity, TgTau^P301L^ mice) and their non-Tg littermates derived by injections into oocytes from 129/SvEvTac × FVB/NJ F_1_ mice and then bred further to obtain congenic derivatives as described previously [13,14]. The Tg line was continuously maintained in a heterozygous state with no use of homozygous breeding stock. For the studies here, back-crosses were continued with 129/SvEvTac mice (N12) to make a congenic derivative. Crosses were also carried out to make a C57BL/6Tac congenic derivative (N11). Animals were maintained in ventilated racks (Tecniplast, Green Line, Toronto, ON, Canada) and fed irradiated chow (LabDiets, 5053, Fort Worth, TX, USA). They were housed with a 12 h/12 h light/dark cycle. Cage environmental enrichment comprised 5 cm diameter plastic tubes and nesting material (“Nestlets”, Ancare Inc., Bellmore, NY, USA). A similar number of Tg animals of both sexes were entered into the experiment (40 males and 52 females for 129SvEv/Tac and 34 males and 34 females for C57BL6/Tac), were assigned to the FMT experiment and allowed to age to the dates indicated hereinafter. All animal experiments were performed in accordance with local (AUP00003969) and Canadian Council on Animal Care ethics guidelines.

### 2.2. Immunohistochemistry

Each brain specimen was fixed by immersion in neutral buffered 10% formalin. Samples were subsequently dehydrated and paraffin-embedded. Six µm sagittal sections were rehydrated, and endogenous peroxidase activity was blocked by treatment with 3% hydrogen peroxide for 5 min. After washes, sections were incubated overnight with tau primary antibody (AT8 Thermofisher, Waltham, MA, USA; 1:200) or Iba1 (WAKO, Osaka, Japan; 1:1000) and visualized with horseradish peroxidase using the DAKO ARK^TM^ kit (Dako, Glostrup, Denmark) following the manufacturer’s instructions. Sections were counterstained using Mayer hematoxylin, dehydrated, and cover-slipped with a permanent mounting medium. Slides were scanned with a NanoZoomer 2.0RS scanner and analyzed using NanoZoomer digital pathology software (NDP.view 2) (Hamamatsu Photonics, Shizuoka, Japan). Digitized slides were then used for quantitative pathology.

### 2.3. Quantitative Pathology

All annotations and quantifications were performed by at least two individuals blind to experimental conditions and sample identities. For quantification of tau pathology, AT8 immunostained sagittal sections were selected to closely match the following coordinates: 0.72 mm lateral to the midline using Paxinos and Franklin’s mouse brain atlas. Standardized quantification was made for the following 12 brain regions: olfactory bulb, cortex, corpus callosum, caudate putamen, nucleus accumbens, hippocampus, thalamus, hypothalamus, midbrain, pons, medulla, and cerebellum. Both astrocytic plaques and neuronal tangles were counted. The values were divided as follows: negative (no staining, <10 (between 1 and 9 plaques or tangles), 10 to 19, 20 to 29, 30 to 39, 40 to 49, 50 to 59, 60 to 69, 80 to 99, 100 to 119, 120 to 139, and 140 to 160 per region. We generated heat maps based on the scores above to assess the spatial distribution of tau immunoreactivity. After the separate brain regions were scored for each mouse, values were imported into Microsoft Excel, where a Vertex 42 heat map program (https://www.vertex42.com/blog/help/excel-help/dynamic-geographic-heat-map-in-excel.html) (accessed on 21 January 2022) was used to generate pathology distribution maps.

### 2.4. RNA Extraction and Nanostring Analysis

Brain tissue (N = 5–7 per pathology class) was frozen in liquid nitrogen and powdered, followed by RNA extraction using the RNeasy Lipid Tissue Mini Kit (Qiagen, Hilden, Germany) and analyzed by Agilent RNA 6000 Nano Kit (Agilent Technologies, Santa Clara, CA, USA) to determine concentration and purity. Then, a multiplex gene expression analysis was performed using the NanoString nCounter^®^ Technology with the Alzheimer’s Disease Panel and nCounter^®^ SPRINT™ Profiler according to the manufacturer protocol (NanoString Technologies, Seattle, WA, USA). Data were analyzed by ROSALIND^®^ (https://rosalind.bio/) (accessed on 28 July 2022), with a HyperScale architecture developed by ROSALIND, Inc. (San Diego, CA, USA). Read Distribution percentages, violin plots, identity heatmaps, and sample MDS plots were generated as part of the QC step. Normalization, fold changes, and *p*-values were calculated using criteria provided by Nanostring (https://www.nanostring.com/) (accessed 18 August 2022). ROSALIND^®^ follows the nCounter^®^ Advanced Analysis protocol of dividing counts within a lane by the geometric mean of the normalizer probes from the same lane. Housekeeping probes to be used for normalization are selected based on the geNorm algorithm as implemented in the NormqPCR R library [24]. The abundance of various cell populations was calculated on ROSALIND using the Nanostring Cell Type Profiling Module. ROSALIND performs a filtering of Cell Type Profiling results to include results that have scores with a *p*-value greater than or equal to 0.05. Fold changes and *p*-values are calculated using the fast method, as described in the nCounter^®^ Advanced Analysis 2.0 User Manual (https://www.nanostring.com/download_file/view/1169/3842) (accessed 27 February 2020). *p*-value adjustment was performed using the Benjamini–Hochberg method of estimating false discovery rates (FDR). Clustering of genes for the final heatmap of differentially expressed genes was performed using the PAM (Partitioning Around Medoids) method using the fpc R library [25] that takes into consideration the direction and type of all signals on a pathway, the position, role, and type of every gene, etc. Hypergeometric distribution was used to analyze the enrichment of pathways, gene ontology, domain structure, and other ontologies. The topGO R library [26] was used to determine local similarities and dependencies between GO terms in order to perform Elim pruning correction. Several database sources were referenced for enrichment analysis, including Interpro [27], NCBI [28], MSigDB [29,30], and REACTOME [31]. Enrichment was calculated relative to a set of background genes relevant for the experiment. A compilation of 23 functional annotations for the Mouse Alzheimer’s Disease panel was used to classify data for differentially expressed genes (DEGs).

### 2.5. DNA Extraction

Mouse feces were collected and immediately frozen on dry ice for storage at −80 °C until use. DNA extraction was performed with QIAamp PowerFecal DNA Kit (Qiagen #12830-50) according to the manufacturer’s instructions, with minor modifications as follows. Tubes were heated for 10 min at 65 °C and simultaneously shaken at 1100 rpm on a thermomixer (Eppendorf, Hamburg, Germany), then vortexed for 10 min at maximum speed (digital vortex mixer, Fisher Scientific, Waltham, MA, USA), and finally, the tubes were placed in the mini bead-beater (Biospeck, Salem, NJ, USA) to optimize the DNA outcome, 3 times for 1 min at maximum speed, with 1 min on ice in each intervening period. The concentration and quality of fecal DNA were measured by NanoDrop™ analysis, in which the absorbance ratios at 260 nm/230 nm and 260 nm/280 nm were determined (around 2 and 1.8, respectively) to evaluate the purity of the extracted DNA. A total of 200 ng of isolated DNA was loaded onto a 1% agarose gel and electrophoresed in 0.5× TAE running buffer (20 mM Tris-acetate/0.5 mM EDTA) to examine the integrity of DNA.

### 2.6. Library Construction and Sequencing

For metagenomics analysis of the mice microbiome, the V3–V4 regions of the 16S rRNA gene were amplified and indexed for next-generation sequencing according to instructions provided by Illumina (San Diego, CA, USA) in [16S Metagenomic Sequencing Library Preparation. Preparing 16S Ribosomal Gene Amplicons for the Illumina MiSeq System. Illumina (https://support.illumina.com/documents/documentation/chemistry_documentation/16s/16s-metagenomic-library-prep-guide-15044223-b.pdf)] (accessed 21 February 2019). Libraries were sequenced on a MiSeq instrument following a 300 bp paired-end protocol, and demultiplexing of samples was performed on-instrument.

### 2.7. Bioinformatics

Base pairs with a quality score (Q) smaller than 24 were trimmed off with fastq-mcf. Subsequent processing of sequences was conducted with the QIIME2 pipeline [32]. Initially, sequences were de-noised using the Deblur algorithm [33], using a trimming length of 220 bp. Representative, de-noised sequences from Deblur were aligned to the most recent version of the Greengenes database [34] using MAFFT [35], and an unrooted phylogenetic tree was built with Fasttree, which was rooted using the midpoint-root function of QIIME2. Phylogenetic analysis was conducted using 10,000 sequences per sample.

For taxonomic classification, a QIIME2 artifact was created with the 99_outs.fa and 99_otu_taxonomy.txt files from Greengenes. The V3-V4 regions of the 16S sequence were cropped from such artifacts. A classifier was built using the fit-classifier-naive-bayes function of QIIME2. Taxonomic classification of representative sequences generated by the Deblur workflow was conducted with the function classify-sklearn of QIIME2.

### 2.8. Colonization of Mice by Fecal Microbiotal Transplant (FMT)

Fresh fecal samples were collected from 550-day-old donor transgenic mice, snap-frozen on dry ice, and stored at −80 °C until use. For 129SvEv/Tac donors, there were 6, 7, and 4 donors in the frontal (F), caudal (C), and pathology-negative (N) categories, and for C57BL/Tac, there were 13, 7, and 8 donors, respectively. In addition, the luminal contents and the epithelial associated microbes of 2 cm portions of the colon, ileum, duodenum, and caecum were collected in sterile PBS containing 50 μg/mL Gentamicin (Merck, Darmstadt, Germany) (the latter to help bacteria detach from the intestinal wall). Samples were centrifuged at 12,000× *g* for 5 min at 4 °C, and the pellets were stored at −80 °C until use. The pooled material for FMT was assembled keeping C57BL6/Tac- and 129SvEv/Tac-derived samples separate (F, C, and N, *n* = 13, 7, and 8 and 6, 7, respectively) and was re-suspended in sterile PBS containing 15% glycerol, 0.05% L-cysteine hydrochloride monohydrate, and 0.5% sodium ascorbate. The slurry was homogenized using a disposable tissue grinder (Covidien, Dublin, Ireland), passed through a 100 µm cell strainer (Corning, Corning, NY, USA), aliquoted, and stored at −80 °C until use. The antibiotic treatment (ATB-tx) protocol was adapted from [36,37] for each background, following the results of pilot analyses. Thus, 180-day-old C57BL/6Tac mice were administered ampicillin 1 g/L (Fisher), neomycin 1 g/L (Fisher), and sucralose 1 g/L (Loblaws, Toronto, ON, Canada) in the daily prepared drinking water, whereas 180-day-old 129/SvEvTac mice received ampicillin 1 g/L, neomycin 1 g/L, metronidazole 1 g/L, and vancomycin 500 mg/L (Alfa Aesar, Ward Hill, MA, USA), and sucralose (0.1 g/L) in the daily prepared drinking water. Water bottles protected from light were monitored to confirm consumption and mice were monitored on a daily basis for signs of dehydration or other sickness. Feces were collected before and after ATB-Tx in order to assess the efficiency of the antibiotic in decolonizing gut microbiota. Depletion of culturable bacteria following antibiotic administration was assessed via standard microbiological culture in aerobic and anaerobic conditions. Aerobic bacteria were grown on MRS Broth (Sigma, St. Louis, MO, USA) or Schaedler blood agar (Fisher). Anaerobic bacteria were grown on blood agar using an anaerobic vented jar [38]. Culturable bacteria counts were obtained by plating serial dilutions of bacteria on the corresponding media for 48 h (aerobes) or 72 h (anaerobes) [39]. Beginning on day 4 of ATB-Tx, mice were recolonized by oral gavage of 200 µL of fecal material (300 mg/mL) once a day for three consecutive days.

### 2.9. Statistical Analyses

For microbial genotyping, differences in Shannon and Simpson diversity between the groups were evaluated by Kruskal–Wallis after checking that data were non-parametric. Data normality and homogeneity of variance were evaluated by D’Agostino and Pearson and Levene’s tests, respectively. Correlations between species at the genus level (primary measure) and the histological classification of 129SvEv/Tac mice (frontal, caudal, pathology-negative, and wild-type) were evaluated using the Spearman rank correlation test. Post hoc analysis was performed using Dunn’s multiple comparisons test. Except for the case of pooled samples for all outcomes where each cell was >5 and assessed by a chi-square test, proportions of expected versus observed pathological outcomes in mouse cohorts were assessed by the Freeman–Halton extension of Fisher’s exact test. Survival curves were assessed by a Mantel–Cox test. Alpha values were set to *p* < 0.05.

## 3. Results

### 3.1. Transcriptional Profiles in TgTau^P301L^ Mice with Frontal or Caudal Tau Deposition

**Figure 1:**
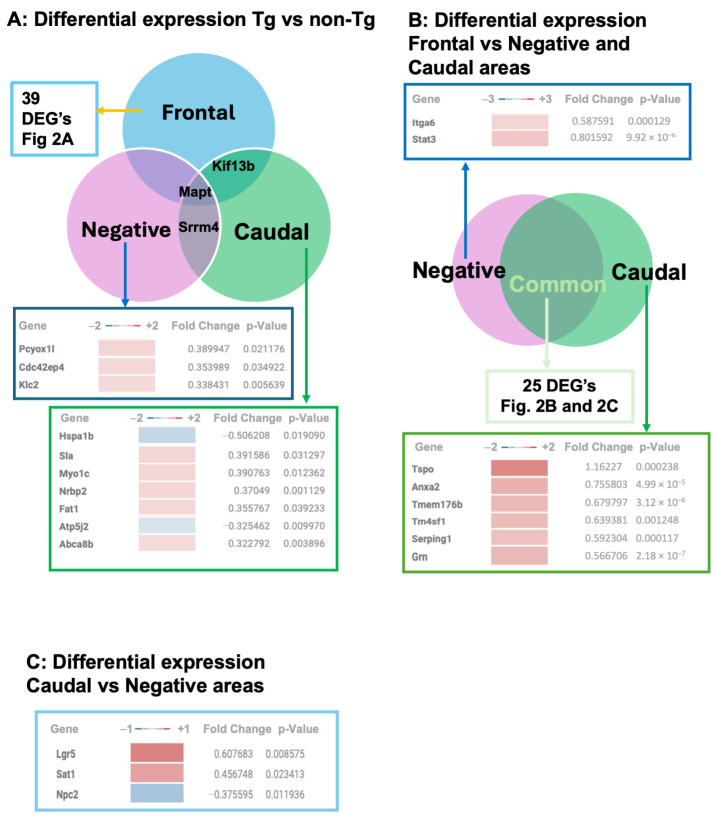
Patterns of altered transcripts in TgTau^P301L^ mice with different brain pathologies. (**A**) Venn diagram comparing the differentially expressed genes in the brain of TgTau^P301L^ mice presenting with frontal (rostral) pathology, caudal pathology, or being scored as pathology-negative at 550 days. Transgenic mice were classified by reactivity with AT8 anti-phospho tau antibody and measured against non-transgenic control mice of equivalent ages. The data identify 3 genes specifically up-regulated versus pathology-negative mice, 5 genes up-regulated and 2 down-regulated in mice with caudal phospho-tau deposition, and (see Figure 2A) 38 genes specifically up-regulated in mice with phospho-tau deposition in frontal areas. Transcripts within overlapping areas of the Venn diagram include *Mapt*, *Kif 13b*, and *Srrm4*. Over-representation of *Mapt* was notably similar in Tg mice classified as frontal, caudal, or pathology-negative (Figure S2A), and the signal was attributed to the mouse *Mapt* nanostring probe cross-hybridizing with transgene-derived human *MAPT* mRNA sequences. In contrast, *Kif 13b* and Srrm4 were not equivalently expressed in frontal, caudal, or pathology-negative Tg animals (Figure S2B,C). *Kif 13b* plays a role in microtubule-based movement and is involved in the reorganization of the cortical cytoskeleton. *Srrm4* enables RNA binding activity and is involved in the proper function of the CNS. (**B**) Venn diagram of differentially expressed transcripts comparing aged TgTauP^301L^ mice with frontal pathology versus pathology-negative transgenic animals or with mice exhibiting caudal pathology. Two transcripts were up-regulated versus the pathology-negative phenotype, and 6 gene transcripts were up-regulated versus the caudal pathology phenotype (see also Figure 2B,C). (**C**) Three genes are differentially expressed in caudal versus no pathology. Twenty-five genes are common to both phenotypes (see also Figure 2B,C).

**Figure 2:**
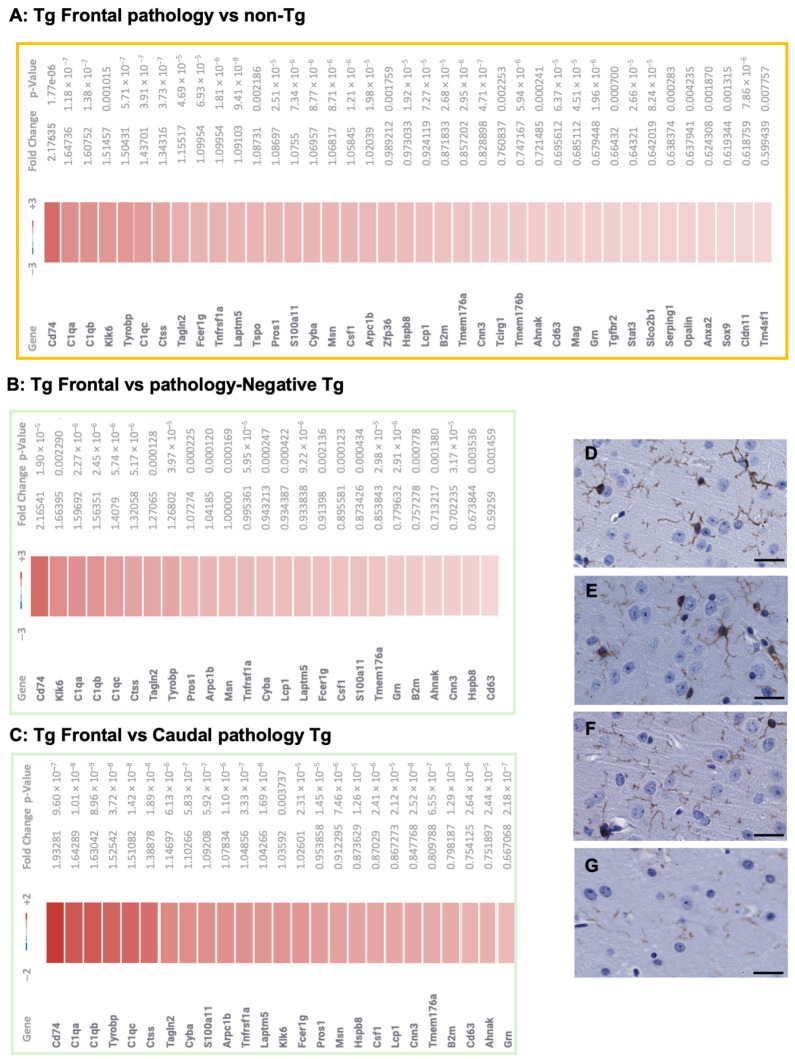
Altered transcripts in TgTau^P301L^ mice and immunohistochemical analysis. Panels (**A**–**C**) present data for up-regulated transcripts in animals with frontal AT8 deposition measured against non-Tg controls (**A**) or against pathology-negative Tg mice (**B**) or against Tg mice with caudal AT8 deposition (**C**). There is an overlap in the transcript identities for panels (**D**–**F**). Numerically, the greatest number of altered transcripts is seen in the comparison versus non-Tg controls (n = 39) and with lower numbers of altered transcripts measured against pathology-negative Tg animals (n = 25) or Tg mice with caudal pathology (n = 25). Presented values represent the Log2 fold changes and *p*-values < 0.05. (**D**–**G**) Corroborating immunohistochemical analyses determined that activated microglia detected with Iba1 antibody were present in aged and young 129SvEv/Tac Tg mice ((**D**,**F**); 553 and 168 d, respectively) as well as their C57BL6/Tac counterparts ((**E**,**G**); 553 and 180 d). Scale bar denotes 25 microns.

To approach the causes of the diversity in tau chemistry and cell biology, we extended prior phenotypic analyses of TgTau^P301L+/−^ congenic mice to include Nanostring transcriptional profiling. Here, to increase statistical power for transcriptional profiling and subsequent analyses, we simplified a prior scheme for dividing the aged cohorts [13] and classified Tg mice into those having rostral versus caudal deposition of tau in the brain or those being pathology-negative for AT8 staining at time of euthanasia. Acknowledging that C57BL6/Tac and 129SevEv/Tac inbred backgrounds had similar heterogeneity of Tau deposition [13], these studies were performed on just one congenic line, namely 129SvEv/Tac TgTau^P301L+/−^ mice. Total RNA was prepared from hemibrains (from 550-day-old animals), probed for integrity by capillary electrophoresis (Agilent RNA 6000), and then analyzed with an Alzheimer’s Disease transcript set encompassing probes for 760 mouse genes. Analyses versus non-Tg controls are presented in Figure 1A. The greatest number of DEGs was scored for mice with frontal pathology, *n* = 39 DEGs, all representing up-regulation (Figure 2A and Figure S1). A total of 7 DEGs were scored for mice with caudal pathology versus 3 DEGs scored in pathology-negative Tg mice, while 3 transcripts in overlapped areas of the Venn diagram are described in Figure S2.

*Comparisons versus non-Tg mice.* The number of scored DEGs in brain RNA offered a parallel to the degree of pathology present in the corresponding hemibrain samples. This perspective was also consistent with other comparisons between the different cohorts of Tg mice; thus, comparing the frontal pathology (F) cohort with either caudal (C) or pathology-negative (N) transgenic groups, the total of DEGs dropped; 25 DEGs were noted in each of the two pairwise comparisons, these being 100% overlapped for transcript identity. All 25 DEGs were represented within the 39 DEGs scored for frontal pathology versus non-Tg controls (Figure 1A, Figure 2B versus Figure 2A and Figure 2C versus Figure 2A). The frontal pathology cohort had 6 DEGs, distinguishing it from the caudal pathology cohort (Figure 1B), but with all 6 transcripts being identified previously (Figure 2A), as was the case for 2 DEGs scored versus pathology-negative Tg mice (Figure 1B). Moving beyond frontal pathology as a point of reference, we turned to comparisons of caudal pathology or pathology-negative Tg mouse brain transcripts versus non-Tg brain transcripts. Here, comparisons between Tg mice with caudal pathology and non-Tg controls (Figure 1A) yielded 7 transcripts with no overlap with the data (Figure 2A–C). Two of these transcripts exhibited down-regulation. Comparing a caudal pathology group to a pathology-negative Tg group yielded 3 DEGs (Figure 1C), again corresponding to transcripts not present in frontal pathology animals (Figure 2A–C). Pathology-negative Tg animals yielded three mildly up-regulated transcripts versus aged non-Tg control samples (Figure S2), these again not being represented as DEGs in frontal pathology animals (Figure 2A–C).

*Pathway analysis in mice with frontal pathology.* Interrelationships between DEGs were next considered in terms of functional networks and cellular lineages. STRING (https://string-db.org) (accessed 2 August 2022) analysis of DEGs from brains with frontal pathology versus caudal pathology or pathology-negative animals or non-Tg controls yielded strong networks with protein/protein interaction (PPI) enrichment (*p*-value: <1.0 × 10^−16^); the most prominent connections involving the 3 complement genes *C1qa*, *C1b*, and *C1qc* (Figures S1A and S3–S5). Considering functional annotations that relate to cell biology, the microglia pathogen phagocytosis pathway from wikipathways (https://www.wikipathways.org) was over-represented in animals presenting a frontal pathology vs. non-Tg animals (*p*-value 3.4 × 10^−5^). This pathway annotation specifies 41 genes, 7 being represented as DEGs in the brains of TgTau^P301L^ mice with a frontal pattern of AT8 phospho tau staining: *C1qa*, *C1qb*, *C1qc*, *Arpc1b*, *Fcer1g*, *Cyba*, and *Tyrobp*. To confirm the striking representation of neuroinflammation-associated transcripts, immunohistochemistry using an Iba1 antibody revealed staining of microglia in 129SvEv/Tac Tg mice, as well as their C57BL6/Tac counterparts measured at different ages (Figure 2D–G). Overall, these data indicate a signal for microglial involvement in neuroinflammatory processes in animals with frontal tau deposition, and as noted by others in analyses of analogous Tg mice maintained on other genetic backgrounds, including C57BL6 [40,41,42,43,44].

*Tg mice with caudal or pathology-negative presentation.* Aged Tg animals with caudal tau deposition or classified as pathology-negative yielded transcript changes that did not overlap with the frontal pathology DEG signature, albeit with smaller magnitudes of absolute changes. Instead, the signature of a distinct set of transcripts was noted (Figure 1B,C). Out of 23 possible functional annotations, a recurrent finding for the 6 transcripts was representation within the cytokine-enriched pathway present for 5 different neuroanatomical classifiers (cerebellum, frontal pole, inferior frontal gyrus, parahippocampal gyrus, and superior temporal gyrus).

*The origins of alternative biological states.* The transcriptomic data presented above define a role for the neuroinflammation processes of the TgTau^P301L^ mice. They also indicate that differing reactive changes may be occurring in Tg mice with different pathology assignments. In terms of the events underpinning different pathology designations, the idea that “F” and “C” represent alternative biological states is supported. At this stage, the overlap in profiles of “C” and “N” mice questions whether the latter really represents a third biological group. Instead, these mice might reflect incomplete penetrance of the transgene and would attain a “C” pathology designation if aged longer. Overall, the presence of neuroinflammatory processes in TgTau^P301L^ mice, yet the restricted genetic variation imparted by the use of congenic stocks, suggested an extrinsic variable as a driver of alternative tau pathology outcomes. Specifically, we considered whether this variable could be equated with genetic diversity in gut microbiota.

### 3.2. 16S rRNA Analysis of Gut Microbiota

**Figure 3:**
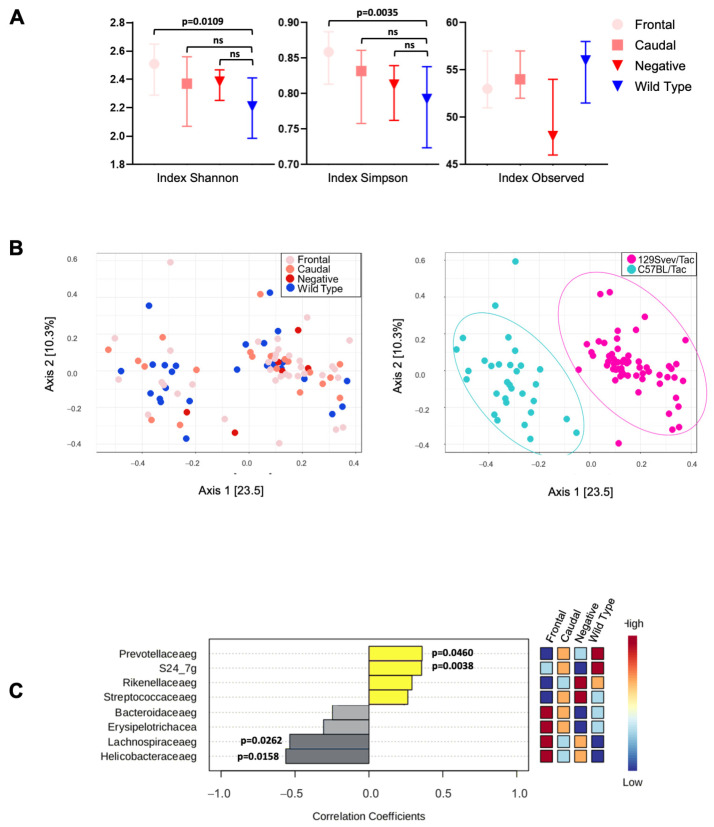
16 rRNA gene analysis from gut-derived C57BL6/Tac and 129Svev/Tac TgTau^P301L^ mouse samples. (**A**) Alpha diversity measured by Simpson, Shannon, and Observed indexes were plotted for frontal, caudal, no pathology, and wild-type mouse groups maintained under basal housing conditions. Values are expressed as median and interquartile range, with significant differences indicated by *p*-value. ns = not significant. (**B**) Scatter plot of principal coordinates analysis (PCOA) showing beta diversity among class (left) and background (right). Differences between groups were tested by a permutation multivariate analysis of variance (PERMANOVA) using a distance matrix function (ADONIS). [PERMANOVA] F-value: 19.332; R-squared: 0.18181; *p*-value < 0.001. (**C**) Representative graph of Spearman’s correlation analysis at the family level. The heat map at right represents the abundance for every taxon. Number of samples, C57BL/Tac = 31, 129Svev/Tac = 58.

**Figure 4:**
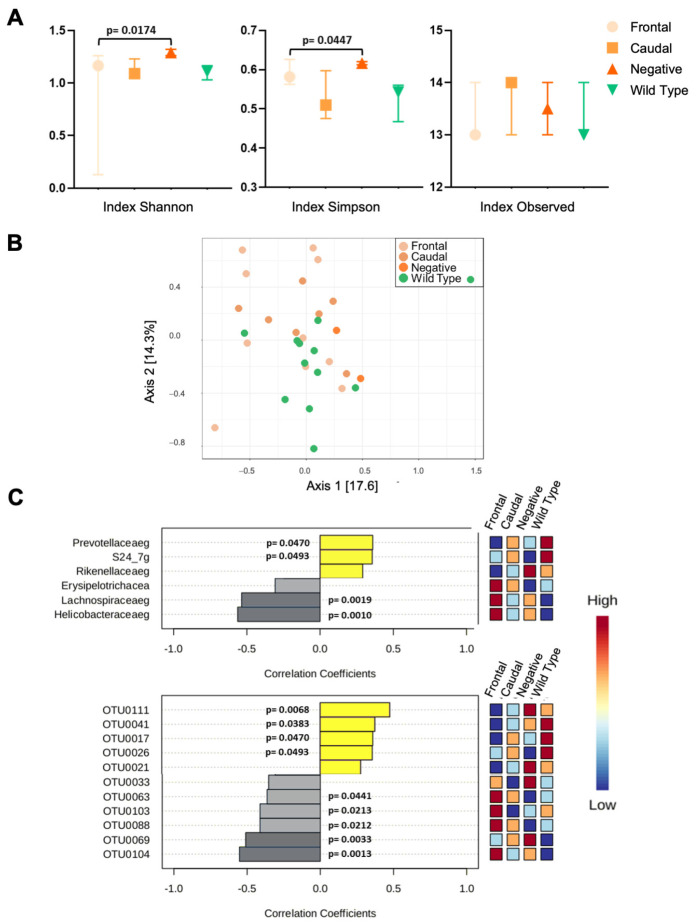
16rRNA analysis from gut-derived C57BL6J/Tac TgTau^P301L^ mouse samples. (**A**) Alpha diversity measured by Simpson, Shannon, and Observed indexes were plotted for frontal, caudal, no pathology, and wild-type groups. Values are expressed as median and interquartile range, with significant differences indicated by *p*-value. (**B**) Scatter plot of principal coordinates analysis (PCOA) showing beta diversity among classes. Differences between groups were tested by a permutation multivariate analysis of variance (PERMANOVA) using distance matrices function (ADONIS), *p*-value < 0.023. (**C**) Representative graph of Spearman’s correlation analysis at family level (upper panel) and OTU level (lower panel). Heat map at right represents the abundance for every taxa number of samples, frontal = 10, caudal = 7, no pathology = 2, and wild-type = 12. Yellow and grey shading indicate, respectively, positive and negative correlation coefficients.

**Figure 5:**
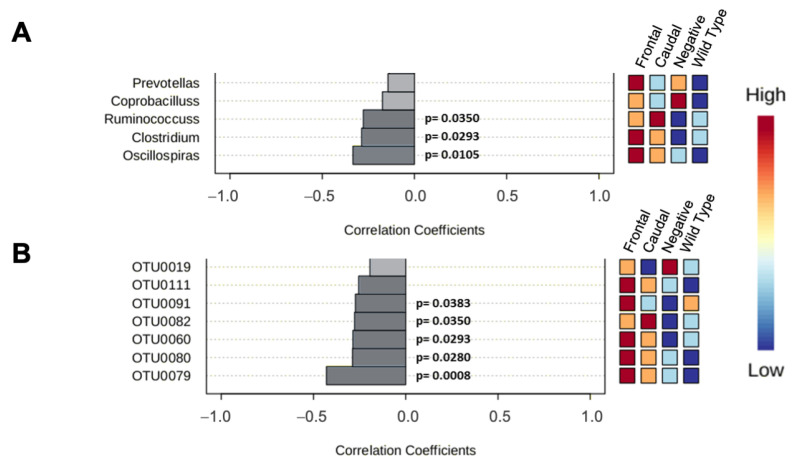
16rRNA analysis from gut-derived 129SvEv/Tac TgTau^P301L^ mouse samples. (**A**,**B**) Representative graph of Spearman’s correlation analysis at genus level (up) and OTU level (down). Heat map at right represents the abundance for every taxon. Number of samples, frontal = 27, caudal = 14, no pathology = 4, and wild-type = 13. Alpha diversity metrics represent the richness and abundance of OTUs within each sample. Several indexes were analyzed at different taxonomic levels. Table S4 shows taxonomic level and diversity measure for 129SvEv/Tac mice samples. No significant difference was found at any taxonomic level. The correlation numbers for *Oscillospiras*, *Clostridium*, and *Ruminococcus* were negatively correlated, showing the lowest expression in the caudal and frontal group (upper panel). The correlation numbers for OTU0079, OTU0080, OTU0060, OTU0082, and OTU0091 were negatively correlated, showing the lowest expression between the groups frontal and caudal (lower panel, Table S5).

For analyses of the gut microbiome, genetic profiling was carried out on rDNA derived from 129SvEv/Tac and C57BL6/Tac TgTau^P301L^ adult mice (>500 d.), with pooled data presented in Figure 3 and C57BL6/Tac and 129 SvEv/Tac data considered separately in Figure 4 and Figure 5, respectively. Within these data, alpha diversity metrics represent the richness and relative abundance of operational taxonomic units (OTUs) within each sample. Shannon, Simpson, and Observed (Figure 3A) index values were used to describe and compare alpha diversity across the sample groups categorized by their pattern of AT8 immunoreactivity and here including animals of both 129SvEv/Tac and C57B6/Tac inbred strain genotypes. The observed species index measures the number of different species per sample, which is defined as “richness”. The relative abundances of the different species making up the samples’ richness are defined as “evenness”. The Shannon and Simpson diversity indexes relate both OTU richness and evenness, while Observed measures only richness.

We measured alpha diversity at different taxonomic levels. Figure 3A shows results at the genus level; however, similar results were found at the family and species level (Table S1). Statistical testing showed no difference for the Observed index (p_Observed_ = 0.082), while the Shannon diversity (p_Shannon_ = 0.0140, Dunn Test *p* < 0.0109) and Simpson diversity indices (p_Simpson_ = 0.0042, Dunn Test *p* < 0.0035) showed greater overall alpha diversity in the frontal group compared to non-Tg (Figure 3A). These data indicate significant differences in gut microbiota between Tg and non-Tg animals in the case of frontal pathology but not in the case of Tg mice that were pathology-negative or had caudal pathology. However, in the case of the Simpson index, there was a trend toward a hierarchy of values going from stronger to weaker pathology.

Bray–Curtis measures for beta diversity were represented by two-dimensional principal coordinates analysis (PCoA) plots (Figure 3B, left panel). Differences between groups were tested by a permutation multivariate analysis of variance (PERMANOVA) using distance matrices function (ADONIS), F-value: 1.9499; R-squared: 0.064389; *p*-value < 0.017. Interestingly, clustering was observed between the samples on the right and left sides of the PCoA graph. We performed another beta diversity analysis for background; the two clusters were found to represent C57BL6/Tac and 129SvEv/Tac mice (Figure 3B, right panel). These data indicate a potent effect of the inbred strain genetic background of the Tg mice, in accord with prior analyses of C57BL6- and 129-related inbred stocks [45]. A Spearman rank correlation test at the family level identified significant changes in *Prevotellaceaeg* and *S24_7g*, while negative correlation coefficients were scores for *Lacchnospiraeaeg* and *Helicobacteraceaeg* (Figure 3C).

#### 3.2.1. 16rRNA from C57BL6/Tac Samples

Several indexes were analyzed at the different taxonomic levels. Table S2 shows taxonomic level and diversity measure values in C57BL6/Tac mice. Phylum, order, and class had a significant difference in alpha diversity. Figure 4A shows the analysis at the class level (taxonomic level) for illustrative purposes. Shannon, Simpson, and Observed index values were used to describe and compare alpha diversity across the sample types by pathology class, with similar results also found at phylum and order taxonomic level (Table S2). Statistical analysis showed no difference for Observed index (*p*_Observed_ = 0.082), while Shannon diversity index (*p*_Shannon_ = 0.0095, Dunn test *p* < 0.0174) and Simpson diversity index (*p*_Simpson_ = 0.0106, Dunn test *p* < 0.0447) showed greater overall alpha diversity in the pathology-negative group compared to the frontal group.

Differences between groups at the class taxonomic level were tested by a permutation multivariate analysis of variance (PERMANOVA) using distance matrices function (ADONIS), F-value: 1.9874; R-squared: 0.18088; *p*-value < 0.023. Our results indicate the existence of beta diversity among the groups. In order to identify the relative abundance of species, we performed a Spearman’s correlation analysis (Figure 4B). Correlations between species at the family level (primary measure) and the histological classification of C57BL6J/Tac mice (frontal, caudal, pathology-negative, and wild-type) were evaluated using the Spearman rank correlation test (Figure 4C). The number of bacteroidetes (*prevotellaceaeg* and *S24_7g*) were positively correlated, showing the highest representation in the wild-type, while the firmicutes (*lachnospiraceae*) and proteobacteria (*helicobacteraceaeg*) were negatively correlated mainly in the frontal group. At the OTU level we observed a positive correlation for OTU0111, OTU0041, OTU0017, and OTU0026 and negative values for OTU0063, OTU0103, OTU0069, and OTU0104 (Figure 4C, lower panel; Table S3).

#### 3.2.2. 16rRNA from 129SvEv/Tac Samples

Table S4 shows taxonomic level and diversity measures for 129SvEv/Tac mice samples. In contrast to the analogous analysis from C57BL6/Tac mice (Table S2), no significant difference was found at any taxonomic level. Correlations between species at the genus level (primary measure) and the histological classification of 129SvEv/Tac mice (frontal, caudal, or pathology-negative Tg versus non-Tg) were evaluated using the Spearman rank correlation test (Figure 5). The correlation numbers for *Oscillospiras*, *Clostridium*, and *Ruminococcus* were negatively correlated, showing the lowest expression in the caudal and frontal groups (Figure 5A). The correlation numbers for OTU0079, OTU0080, OTU0060, OTU0082, and OTU0091 were significantly negatively correlated, showing the lowest expression between the frontal and caudal groups (Figure 5B; Table S5). Notably, the species and OTUs identified here as having significant correlation changes did not overlap with those identified as correlated in C57BL6/Tac samples, with the sole exception of *prevotellas*, which yielded a negative value for correlation (n.s.) versus a positive correlation value in C57BL6/Tac mice (*p* = 0.047).

### 3.3. Pathology Outcomes After Microbial Transplantation

**Figure 6:**
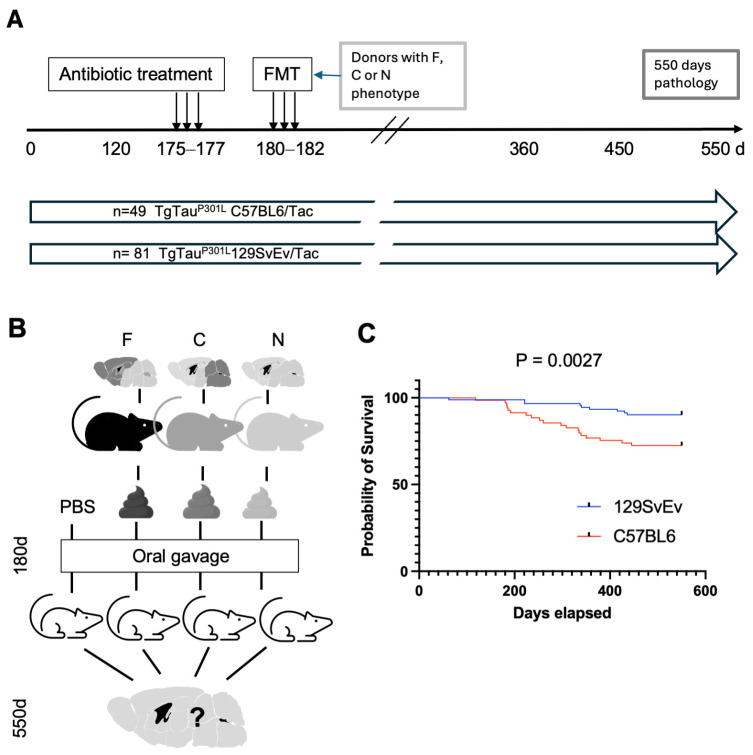
Experimental design and cohorts used for fecal microbial transplantation performed in TgTau^P301L^ mice. (**A**) Treatment regimen for the transplantation experiment with timeline shown left to right in days (not to scale). Sample sizes for cohorts of C57BL6/Tac and 129SvEv/Tac congenic TgTau^P301L^ mice completing the experiment are shown. Treatment steps are denoted by vertical arrows. (**B**) Origin of fecal samples and elaboration of the treatment (see also (**A**)). Question mark denotes that the outcomes of different gavage manipulations were unknown in advance of the experimental manipulations. (**C**) Age-related attrition in the cohorts of C57BL6/Tac and 129SvEv/Tac congenic TgTau^P301L^ mice. Differing results in the two inbred backgrounds were highly significant (*p* = 0.0027).

To go beyond correlative analyses in aged animals, we next set out to manipulate microbial populations. Four treatment regimens were applied to young Tg mice: (i) (ATB-Tx) alone with administration of gavaged diluent (a saline-based solution), or (ATB-Tx) followed by gavage with microbial preparations from aged Tg mice classified as having (ii) frontal pathology (“F”), or (iii) caudal pathology (“C”) or (iv) pathology-negative (“N”; Figure 6A). ATB-Tx for 72 h of water intake showed depletion of bacteria (99%) in comparison with samples before the antibiotic treatment (Non-ATB-Tx) in De Man, Rogosa, and Sharpe agar (MRS) for Lactobacilli media, Schaedler blood agar in aerobic and anaerobic conditions, *n* = 9/per group. Figure S6 represents results from transgenic groups. Subsequent to ATB-Tx, animals were gavaged with different preparations of fecal slurries re-suspended in a buffer [36]. Then, after a waiting period of 375 d., Tg mice of both genetic backgrounds were euthanized and scored for the pattern of CNS tau deposition (Figure 6A,B). Over the 18-month course of the experiment, 92 animals entering the 129SvEv/Tac arm of the experiment (40 M, 52 F) experienced 9 intercurrent deaths, and 68 animals entering the C57BL6/Tac arm of the experiment (34 M, 34 F) experienced 19 intercurrent deaths. Thus, intercurrent deaths across the whole study totaled 10% in the 129SvEv/Tac background (n = 3 M, 6 F) and 28% in the C57BL6/Tac background (n = 8 M, 11 F), a highly significant result (Figure 6C), and there was a skew for more female intercurrent losses in both cases. A striking asymmetry was noted for losses in the 129SvEv/Tac cohort receiving the caudal slurry preparation, where 50% of animals were affected, irrespective of sex.

**Figure 7:**
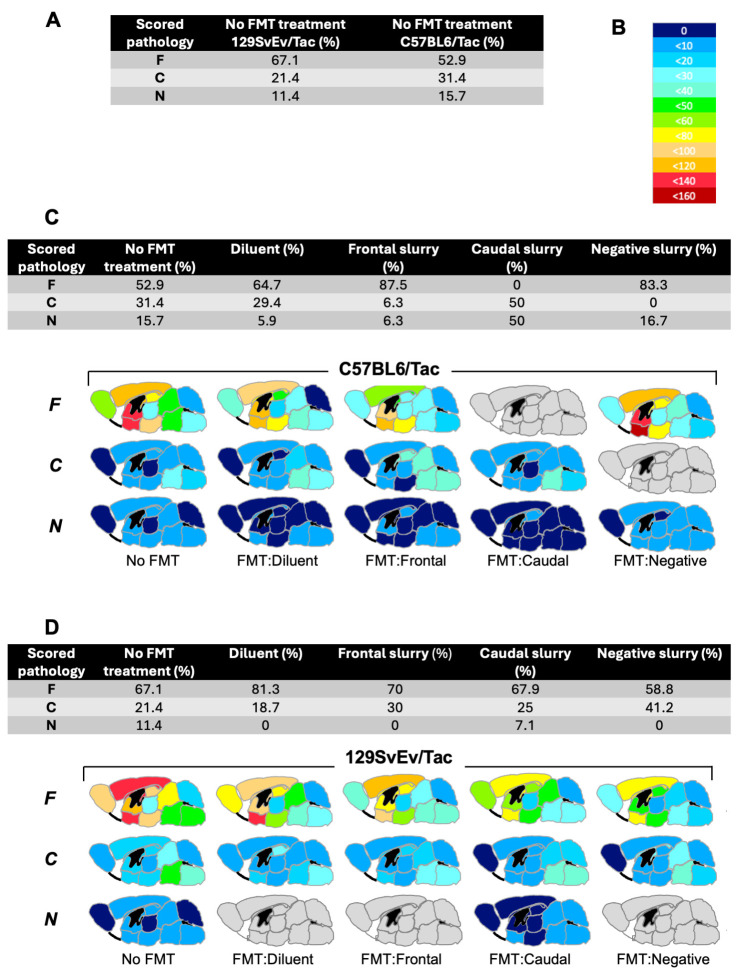
Pathology outcome measures for fecal microbial transplantation performed in TgTau^P301L^ mice. (**A**) Testing differences between the two lines of Tg mice under basal housing conditions. F, C, and N denote frontal pathology, caudal pathology, and pathology-negative status. Untreated C57BL6/Tac Tg mice did not differ in their proportions of F, C, and N outcomes versus their 129SvEv/Tac counterparts (chi-square 2.015, *p* = 0.365, 2 degrees of freedom). To aid group comparisons, these data are repeated in the leftmost columns of panels (**C**,**D**). (**B**) Heatmap legend for AT8 antibody immunostaining data presented in panels (**C**,**D**). (**C**) Results of transplantation in C57BL6/Tac TgTau^P301L^ mice. Treatment groups are shown left to right. FMT = Fecal microbial transplantation. Mice were treated with antibiotics and later gavaged with diluent or gavaged with fecal preparations harvested from aged Tg mice of the designated pathology groups: frontal, caudal, and negative. Observed outcomes of pathology categories measured at 550 days are shown on the vertical axis. Heat maps show the density of tau deposits in different anatomical areas as per panel (**B**). Brain profiles filled in grey denote that animals were not obtained in this category. The table above the brain maps shows the breakdown of data for transplantation outcomes (F, C, N) as percent values, with untreated mice “no FMT treatment” serving as a point of reference. Sample sizes from left to right were 51, 17, 16, 4, and 12. See the main text for statistical analysis of group comparisons. (**D**) Analogous transplantation experiment performed for 129SvEv/Tac Tg mice with treatment groups and outcome categories presented as in panel (**C**). Sample sizes from left to right were 70, 16, 20, 28, and 17.

We next compared patterns of tau deposition in the cohorts of treated mice versus a sample set of untreated animals (Figure 7). For untreated mice, the numbers were 47, 15, and 8, respectively, for F, C, and N categories for 129SvEv/Tac and *n* = 27, 16, and 8 for C57BL6/Tac; here, C57BL6/Tac mice did not differ significantly from their 129SvEv/Tac counterparts (chi-square 2.015, *p* = 0.365) (Figure 7A). We note that two 129SvEv/Tac animals completing the experiment were not entered into histological analysis due to sample preparation issues. Overall, these data for untreated mice align with analyses of prior cohorts, which noted a preponderance of frontal pathology irrespective of these two inbred strain backgrounds [13]. Concerning administration of antibiotic and gavage with diluent (regimen i), neither 129SvEv/Tac Tg mice nor C57BL6/Tac Tg mice differed significantly from their untreated control groups in the proportions of patterns of AT8 positive tau deposited in aged mice (i.e., categories F, C, or N); exact test *p* = 0.57 and *p* = 0.70, respectively (lower panels in Figure 7C,D).

We next considered whether gavage with fecal material might impact brain pathology outcomes measured 15 months later, with heatmap representations of outcomes shown in Figure 7C,D (upper panels; scoring legend, Figure 7B). Considered overall, 65 129SvEv/Tac Tg mice in treatment categories (ii), (iii), and (iv) did not differ significantly from 16 animals in treatment group (i), nor from 70 untreated animals of the same genetic background (*p* = 0.60; 0.13, respectively). Similar findings were noted for C57BL6/Tac Tg mice in treatment categories (ii), (iii), and (iv), where 49 slurry-treated mice did not differ significantly in proportions from the 16 animals in treatment group (i), or 51 untreated C57BL6/Tac Tg animals (*p* = 0.18, 0.58, respectively). Since neither the 129SvEv/Tac nor the C57BL6/Tac background indicated a statistically significant effect of transplantation in these internal comparisons, a fully penetrant, one-to-one correspondence between the type of gavage and pathology outcome is not supported. However, outcomes in the two genetic backgrounds diverged in different directions from a baseline predicted by the controls in treatment group (i). Considering the frontal category of pathology with the largest sample sizes, trends for the largest discrepancies diverged between 129SvEv/Tac and C57BL6/Tac Tg mice. For example, a surfeit of observed frontal pathology in groups (ii) and (iv) in the C57BL6/Tac Tg mice (n = 24 total observed versus 14 total expected) was not scored in 129SvEv/Tac Tg mice (n = 24, 24 observed and expected; Figure 7C,D). A post hoc test was used to compare observed outcome measures in treatment groups (ii)–(iv) to ascertain possible differences between the two strain backgrounds. In this analysis, observed pathological outcomes were markedly divergent in C57BL6/Tac versus 129SvEv/Tac Tg mice (n = 14, 0, and 10 F, C, or N pathologies versus *n* = 14, 19, 10, respectively; *p*= 0.0001), but also noting that the highest levels of intercurrent losses affected sample sizes in the caudal treatment group of C57BL6/Tac mice.

## 4. Discussion

### 4.1. Phenotypic Variation Within Models of FTLD-MAPT

Previous analyses have documented diverse forms of tau deposition in different brain regions of aged TgTau^P301L^ mice using the AT8 antibody commonly applied for staging of brain material from tauopathy patients [13]. Using techniques to probe tau epitopes in denatured brain samples, diversity was also noted in tau conformer ensembles in these P301L transgenic mice, as well as in human FTLD-MAPT-P301L brain samples [16]. Diversity in phenotypic parameters is apparent in between Tg models and mice made with a Thy-1 neuronal promoter and harboring a P301S mutation (Tg2541; [46]) have prominent tau deposition in the brain stem, not frontal areas, a result that has been equated with variable levels of expression of the sortilin protein [47]. Cell biology adds another level of complexity, noting that disease-associated tau is not restricted to neurons in human 4R tauopathies but is also present in astrocytes and oligodendrocytes [17,48]. Tau deposition in different lineages can also be manifested in animal models with spontaneous disease, e.g., TgTauP^301L^ mice used here [14], or with disease triggered by injection of seed-competent brain material [49]. Data presented here confirm and extend the observation that frontal pathology is more prevalent than caudal pathology in untreated TgTau^P301L^ mice [13] and that the disease process in these animals is associated with activated microglia, even at an early age (Figure 2). Moreover, transcriptional analysis suggests the possibility of different cell biological processes occurring in mice with different pathology classes, rather than a single pathogenic process. The question then arises as to the operational parameters and possible environmental influences relevant to these processes. Because diversity of tau pathologies and disease-associated conformers in TgTau^P301L^ mice occurred irrespective of uniform pan-neuronal expression of the human tau transgene in young mice and use of inbred strain backgrounds [13,16], genetic modifier loci (also known as quantitative trait loci) are likely irrelevant. On the other hand, determinants of relevance to neurodegenerative diseases might reside within the genomes of resident microbiota. In this vein, while there is extensive literature on microbiota in the context of Parkinson’s disease [19,20], less is known in the context of ADRD. We, therefore, focused on this knowledge gap with the experimental design shown in Figure 6.

### 4.2. Microbial Profiles and Phenotypic Variation in a Genetic Tauopathy

Functional relationships between ensembles of gut microbiota and patterns of CNS tau deposition might exist in two forms. A pathology-centric view is that different tau CNS pathologies (F, C, and N) determine correspondingly different gut microbiomes, while a microbiome-centric view would invert this relationship. We also considered whether the data to support functional relationships are insufficient or underpowered, and a null hypothesis that gut microbial genotypes are unconnected to the manifestation of CNS pathologies.

Functional interrelationships were first explored by comparing genetic profiles of gut microbiota in untreated, aged animals. Some differences were noted between mice of different pathology classifications in principal component analyses, but these effects were overshadowed by differences between the two genetic backgrounds (Figure 3B). Given that 129SvEv/Tac and C57BL6/Tac TgTau^P301L^ mice have similar proportions of F, C, and N pathologies (Figure 5 and Figure 7A) in the face of markedly different microbiota, the notion of a simple one-to-one correspondence between a particular microbial species and a particular pathology outcome is not well supported. However, there are two qualifications. First, the microbial composition profiled at the experimental endpoint could be uninformative if deterministic events occur prior to adulthood. Second, thresholds for genotyping sensitivity may limit the ability to detect low-abundance microbes that might influence scored pathology. In practice, our analyses revealed changes at the family level (*Prevotellaceae*, *Lachnospiracae*, *Helicobacteraceae*, and *S24.7*) and for specific OTUs between the four experimental groups. These pooled results constitute evidence of a differential microbiome profile among the pathology-positive, pathology-negative, and wild-type groups (Figure 3 and Figure 4), with changes in the C57BL6/Tac mice being more notable than those in 129SvEv/Tac mice (Figure 4 and Figure 5). Changes in *Helicobacteraceae* abundance were reported in the APPswe/PS1dE9 mouse model of Alzheimer’s disease [50] and in 3xTg mice (expressing APP, PS1, and P301L tau) have similar results in abundance for the family taxa *S24.7* [51].

Going beyond correlative studies, transplantation experiments used broad-spectrum antibiotic interventions to deplete gut microbiota, followed by the introduction of fecal suspensions. Phenotypic profiling was then performed one year later (Figure 6A). Considering the putative effect of ATB-Tx used for the gavage on ADRD pathologies, it has been shown that antibiotics have an effect on amyloidosis in Alzheimer’s model mice [52,53]. Our studies, focusing upon tau as an aggregating protein species, assessed the effects of potent ATB-Tx regimens in two genetic backgrounds, but in neither case were outcomes from cohorts receiving ATB-Tx and diluent gavage different from analyses of aged, untreated animals (Figure 5). This result stands in contrast to effects noted in P301S mice [54] maintained on a Cg C57BL6 background, albeit with an ApoE3 knock-in allele [55]. Furthermore, no differences were noted for outcomes scored in antibiotic-treated male versus female mice. Regarding transplantation studies where ATB-Tx was followed by gavage of fecal slurries from aged animals, there were instances where proportions of AT8-positive CNS pathologies in recipient congenic TgTau^P301L^ mice differed from baseline. In these studies, the degree and direction of alterations in proportions differed in two inbred strain backgrounds, with measured deviations from a starting baseline common to the two inbred strains being more notable in treated C57BL6/Tac TgTau^P301L^ animals. Our analyses revealed a significant difference in the overall proportions of the most abundant frontal pathology outcome between the two genetic backgrounds (*p* = 0.0001) but do not yet support significant category-by-category distinctions within a genetic background. Future studies will need to accommodate different levels of frailty. Another issue complicating head-to-head comparisons is that the antibiotic regimens needed to reduce the counts of plated gut bacteria differed for the two inbred strains; based upon pilot experiments, instances of dehydration and dramatic weight loss in the C57BL6/Tac mice were attributed to the inclusion of metronidazole and vancomycin at dosages of 0.5 or 1.0 g/l. We accommodated this effect by eliminating these two antibiotics from the ATB-Tx treatment cocktail applied to the C57BL6/Tac mice. While this simpler cocktail still reduced the types of bacteria compatible with our plating assays, over the full term of the experiment, it is possible that 129SvEv/Tac mice receiving the complete cocktail were more depleted for resident microbiota and hence better colonized than C57BL6/Tac mice. However, new studies would benefit from additional analyses at the experimental endpoint. First, microbial genotyping to determine the depth of recolonization and tracking of metabolites that might trace alterations in gut/brain communication. Second, spatial transcriptomics on animals with F, C, or N outcomes. These approaches might allow the exploration of hypotheses implicit within the current data, for example, the ability of microbiota derived from “frontal” mice to promulgate the corresponding pathology in transplantation recipients. Reciprocally, finding microbiota that block CNS tau pathologies would be of immense practical use. This protective action has been described by others [55,56], but in this instance is not an attribute of gut microbiota derived from pathology-negative Tg mice.

### 4.3. Mouse Inbred Strains and Modeling of Protein Folding Diseases

This work was prompted by the notion that environmental inputs might affect CNS phenotypes in the Tg line first denoted Tg23027, a line marked by remarkably stable steady-state expression from transgene array measured over the course of a decade [13,14]. While aged C57BL6/Tac and 129SvEv/Tac mice TgTau^P301L^ have a similar repertoire of pathological presentation [13], a central finding of this work is the influence of inbred strain type. In the experiments here, C57BL6/Tac TgTau^P301L^ mice appeared more susceptible to an experimental perturbation and to have greater frailty. Although not commented upon in a prior, smaller study [13], the finding here was highly significant (*p* = 0.0027). Notable divergence in the probability of survival commenced at around the time of the gavage treatment, with 5/19 losses in the C57BL6/Tac TgTau^P301L^ mice at this time versus 0 in 129SvEv/Tac mice TgTau^P301L^. Considering events after 200 d, there was over twice as much intercurrent loss within the C57BL6/Tac axis of the study (21% vs. 9%). Of the C57BL6/Tac animals found dead and with brain tissue processed for pathological analysis, 7/8 were pathology-negative (range 180–350 days). While the data from these 7 animals are unsurprising, given the slow onset of pathology in this model [13], they speak to the idea that intercurrent losses are not, per se, linked to the deposition of phosphotau species visible by light microscopy. Instead, this phenomenon calls to mind well-documented, acute effects for the generation of Tg mice expressing the 695 amino acid isoform of amyloid precursor protein (APP), where increasing the percentage of C57BL6 background reduced viability and, in some cases, confounded the creation of congenic derivative lines [57,58]. This phenomenon, encountered at the dawn of Tg studies of AD, had a practical impact on the generation of mouse models of other neurodegenerative diseases; founder lines were often perpetuated in specific C57 sublines or in hybrid C57BL6 backgrounds or in non-C57BL6 backgrounds [14,54,59,60,61]. This approach was followed in spite of a pure C57BL6 background being favored for neuroscience studies, supported by superior levels of hippocampal long-term potentiation and performance in behavioral tasks [62]. In the case of APP, endothelial dysfunction was implicated as a contributing factor for frailty in C57BL6 backgrounds [63,64], and it is possible that more general reactions of the CNS to disease (e.g., neuroinflammatory responses), rather than the effects of particular protein fibril conformation, might underlie a negative synergism with a C57BL6 background. From a practical perspective, the phenomenon begs the question of whether other inbred strain backgrounds—often with larger litters and less susceptibility to loss of pups in early life [65,66,67]—might be worthy of consideration for studies where the favored endpoints are molecular biological and biochemical readouts. In a similar vein, pre-clinical studies that aim to score the effects of candidate therapeutics might benefit from assessments in different mouse inbred strain backgrounds to assess robustness and to serve as a partial proxy for human genetic diversity [68,69].

## 5. Conclusions

Prompted by the analysis of congenic sublines and neuroinflammatory signatures in a transcriptomic analysis, we questioned whether heterogeneous gut microbe populations were driving heterogeneities in pathology and chemistry in TgTau^P301L^ mice. Within an inbred strain background, aged mice with F, C, or N pathology presentations exhibited some distinct gut genotypic profiles, yet with comparisons between C57BL6/Tac and 129SvEv/Tac sublines yielding stronger effects (*p* < 0.001). Divergence between the two inbred sublines was noted in pathology outcomes following FMT with bacterial slurries (*p* = 0.0001) and also in responses to antibiotic treatment or in frailty, measured until the age of 18 months (*p* = 0.0027). Data in the C57BL6/Tac subline indicate a mild ability of the FMT transplants derived from F and C mice to perturb the presentation of frontal or caudal tau deposition in recipients. Conversely, the hypothesis that transplants from N mice suppress tau pathology is rejected, as measured in either C57BL6/Tac or 129SvEv/Tac subline of TgTau^P301L^ mice. Further validation of the effects of FMT awaits experimental refinements suggested by the current study.

## Data Availability

The original contributions presented in this study are included in the article/Supplementary Materials. Further inquiries can be directed to the corresponding authors.

## Supplementary Materials

The following supporting information can be downloaded at: https://www.mdpi.com/article/10.3390/biom15050636/s1. Figure S1: Microglia Pathogen Phagocytosis Pathway; Figure S2: Genes overexpressed in TgTauP301L vs. non-Tg in common; Figure S3: TgTauP301L with frontal pathology vs non-Tg; Figure S4: TgTauP301L with frontal pathology vs caudal pathology; Figure S5: TgTauP301L with frontal pathology vs negative pathology; Figure S6: Bacteria cultivation; Table S1. Alpha diversity analysis at different taxonomic levels for 12Svev/Tac and C57BL/Tac mice samples; Table S2. Alpha diversity analysis at different taxonomic levels for C57BL/Tac mice samples; Table S3. Taxonomic description of selected OTU from Figure 3C; Table S4. Alpha diversity analysis at different taxonomic levels for samples of 129SvEv /Tac mice; Table S5. Taxonomic description of selected OTU from Figure 3 down.
