# Supplementary material for: Microbial Composition, Disease Trajectory and Genetic Background in a Slow Onset Model of Frontotemporal Lobar Degeneration
Source: Biomolecules. 2025 Apr 29;15(5):636. doi: 10.3390/biom15050636 (PMC12109532; doi:10.3390/biom15050636)
Supplement: Supplementary file 1 [file biomolecules-15-00636-s001.zip › biomolecules-3505170-supplementary.pdf]

Figure S1: Microglia Pathogen Phagocytosis Pathway

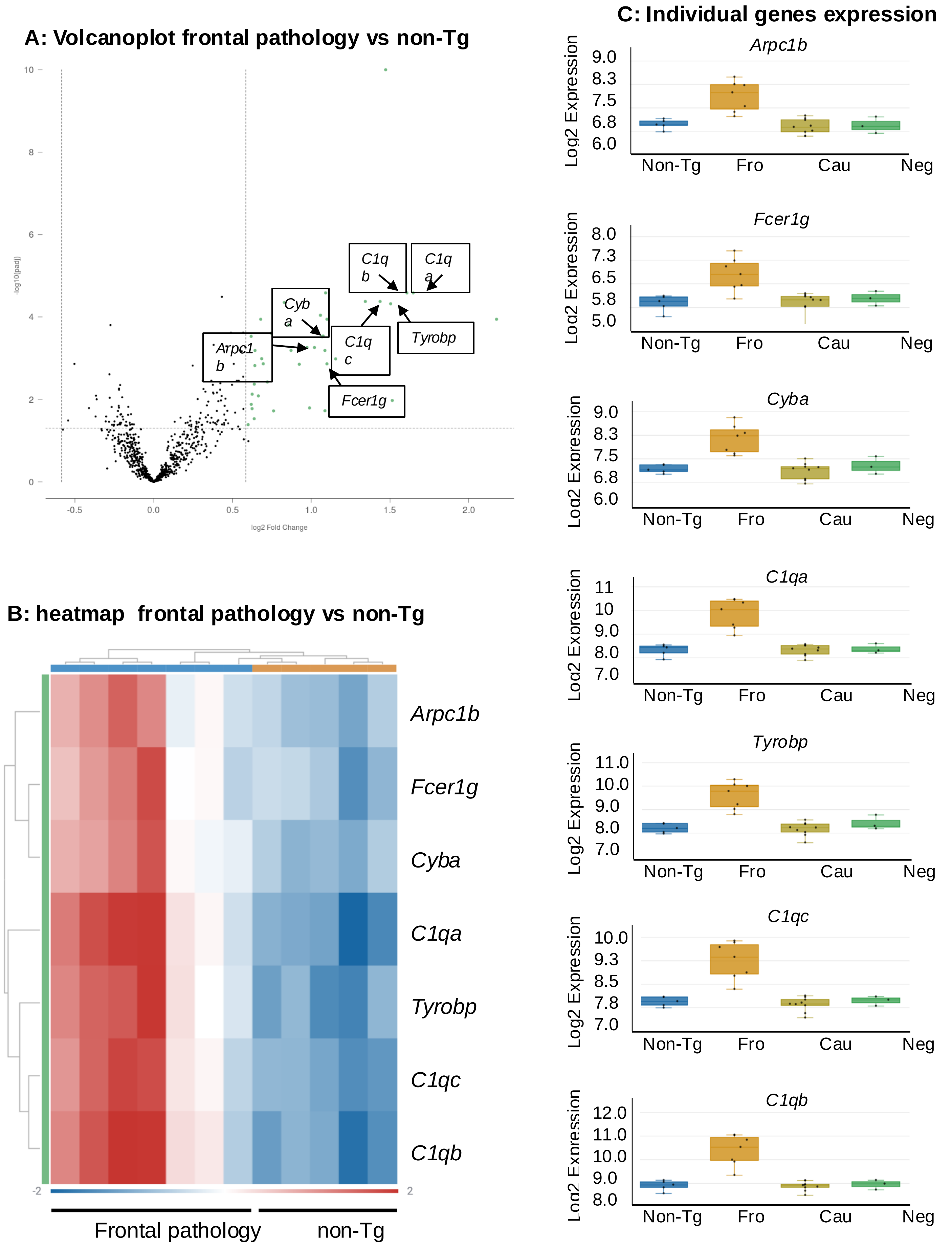

Figure S2: Genes overexpressed in TgTau^{P301L} vs non-Tg in common

A: *Mapt*

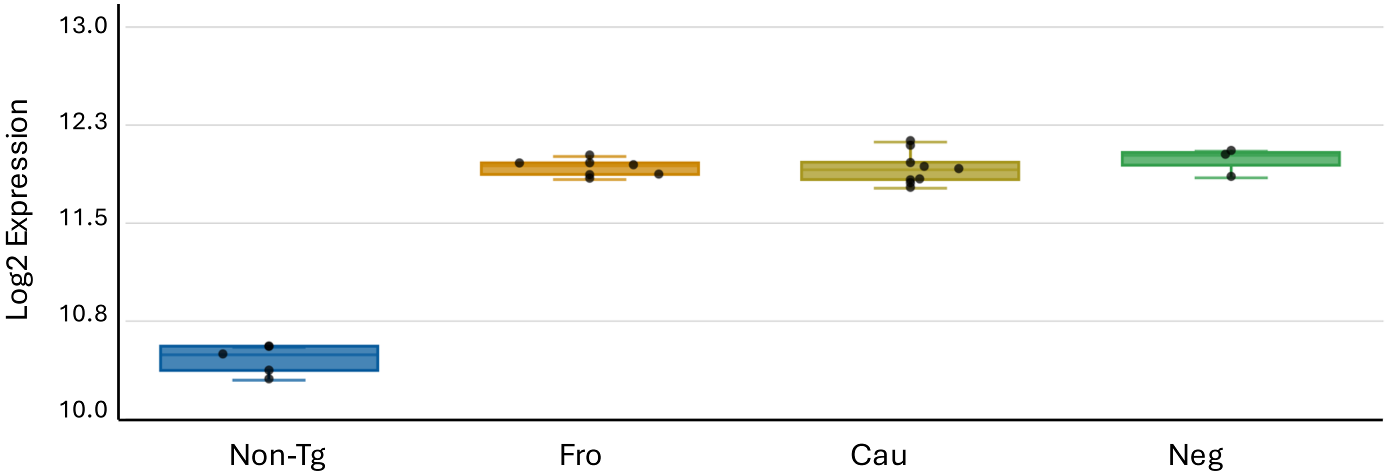

B: *Kif13b*

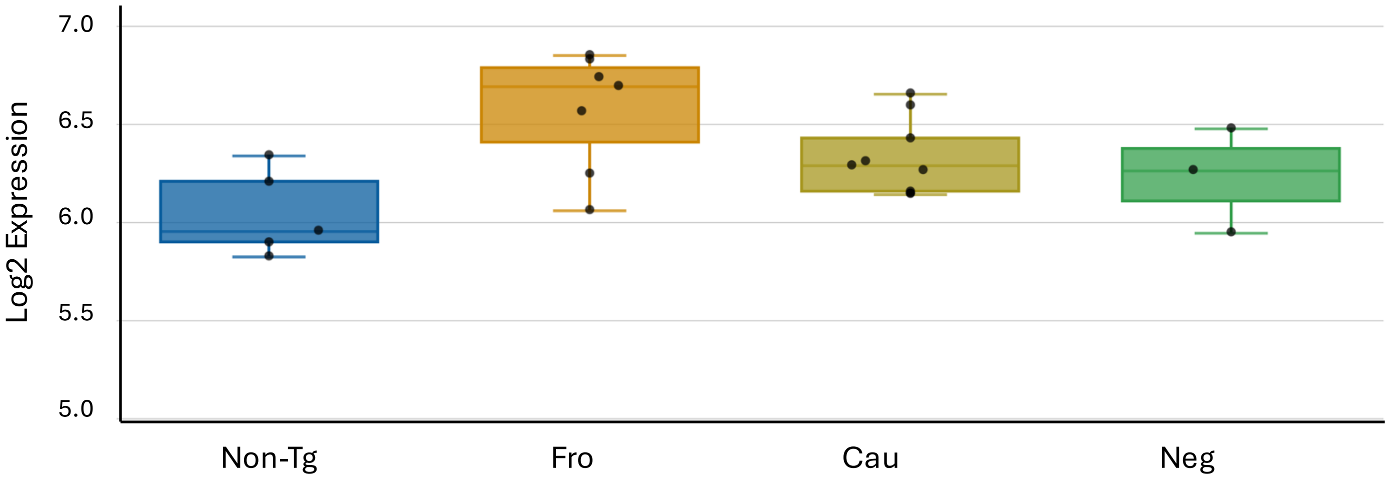

C: *Srrm4*

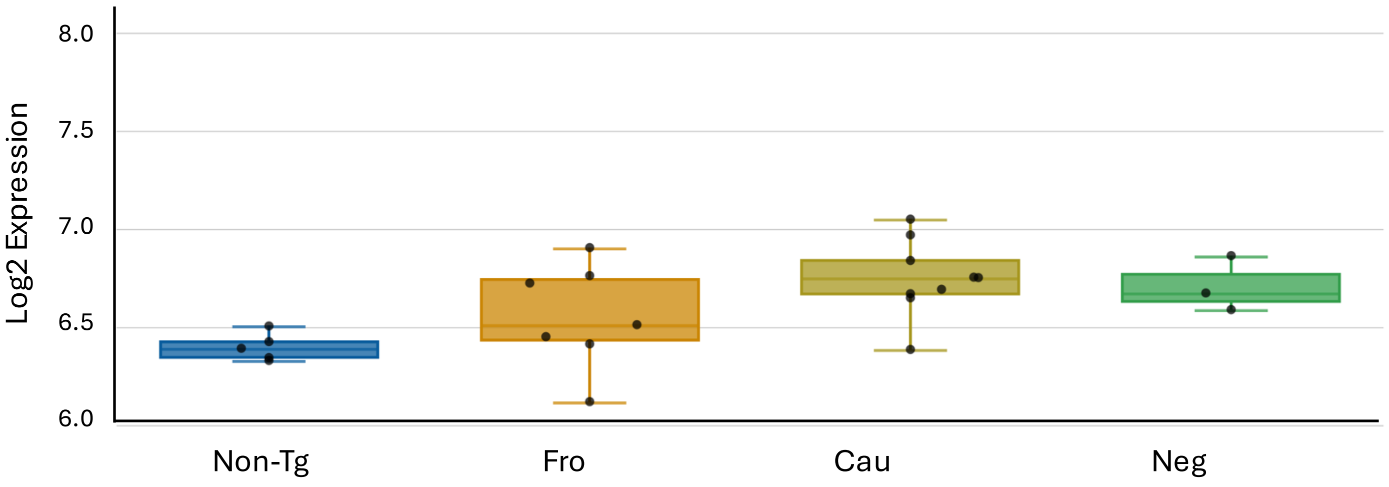

Figure S3: TgTau^{P301L} with frontal pathology vs non-Tg

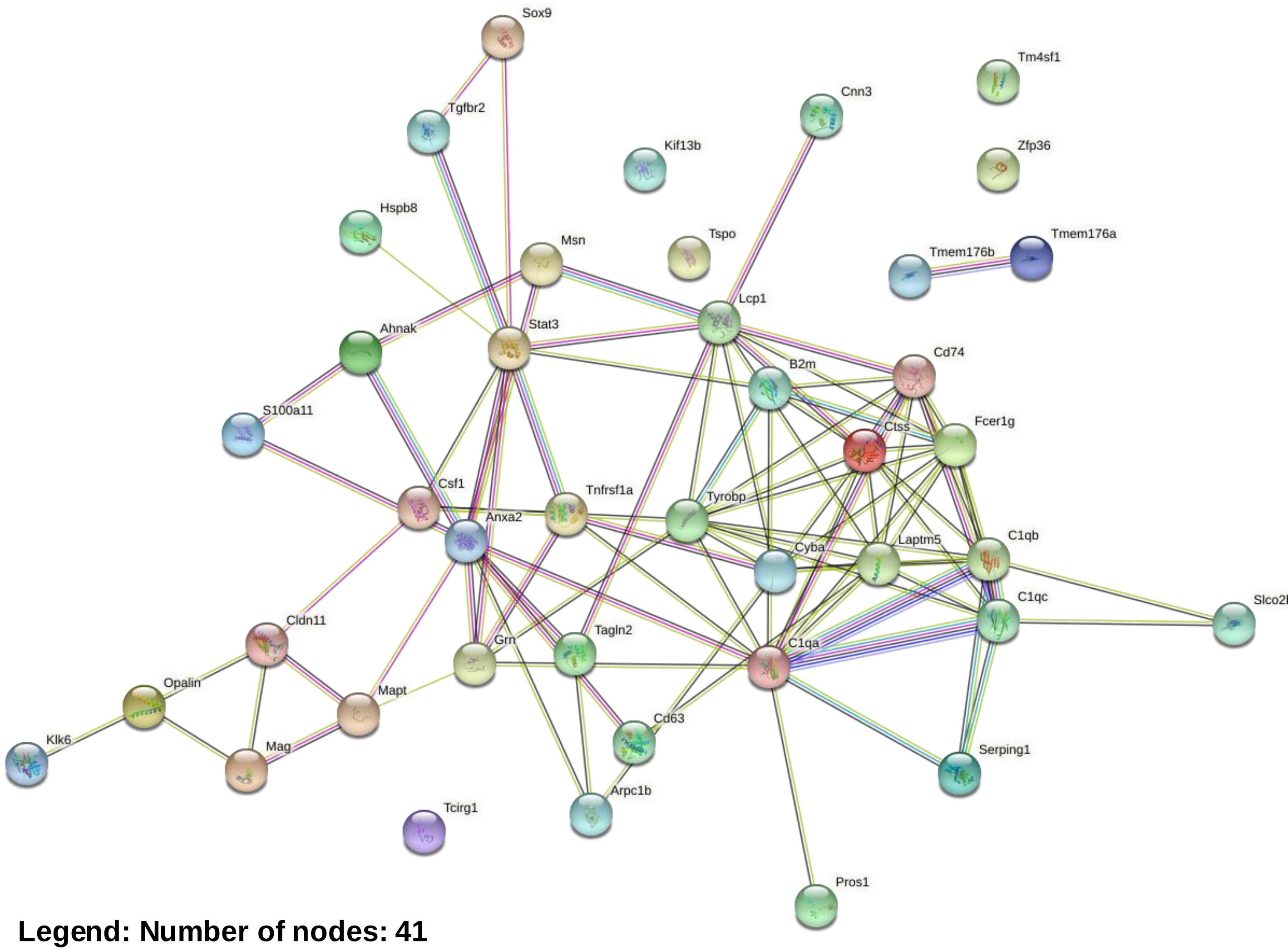

Legend: Number of nodes: 41
Number of edges: 93
Average node degree: 4.54
Avg. local clustering coefficient: 0.539
Expected number of edges: 15
Protein-protein interaction (PPI) enrichment p-value: < 1.0e-16

Nodes:

Network nodes represent proteins

splice isoforms or post-translational modifications are collapsed, i.e. each node represents all the proteins produced by a single, protein-coding gene locus.

Node Color

colored nodes: query proteins and first shell of interactors

white nodes: second shell of interactors

Node Content

empty nodes: proteins of unknown 3D structure

filled nodes: some 3D structure is known or predicted

Edges:

Edges represent protein-protein associations

associations are meant to be specific and meaningful, i.e. proteins jointly contribute to a shared function; this does not necessarily mean they are physically binding to each other.

Known Interactions

from curated databases

experimentally determined

Predicted Interactions

gene neighborhood

gene fusions

gene co-occurrence

Others

textmining

co-expression

protein homology

Figure S4: TgTau^{P301L} with frontal pathology vs caudal pathology

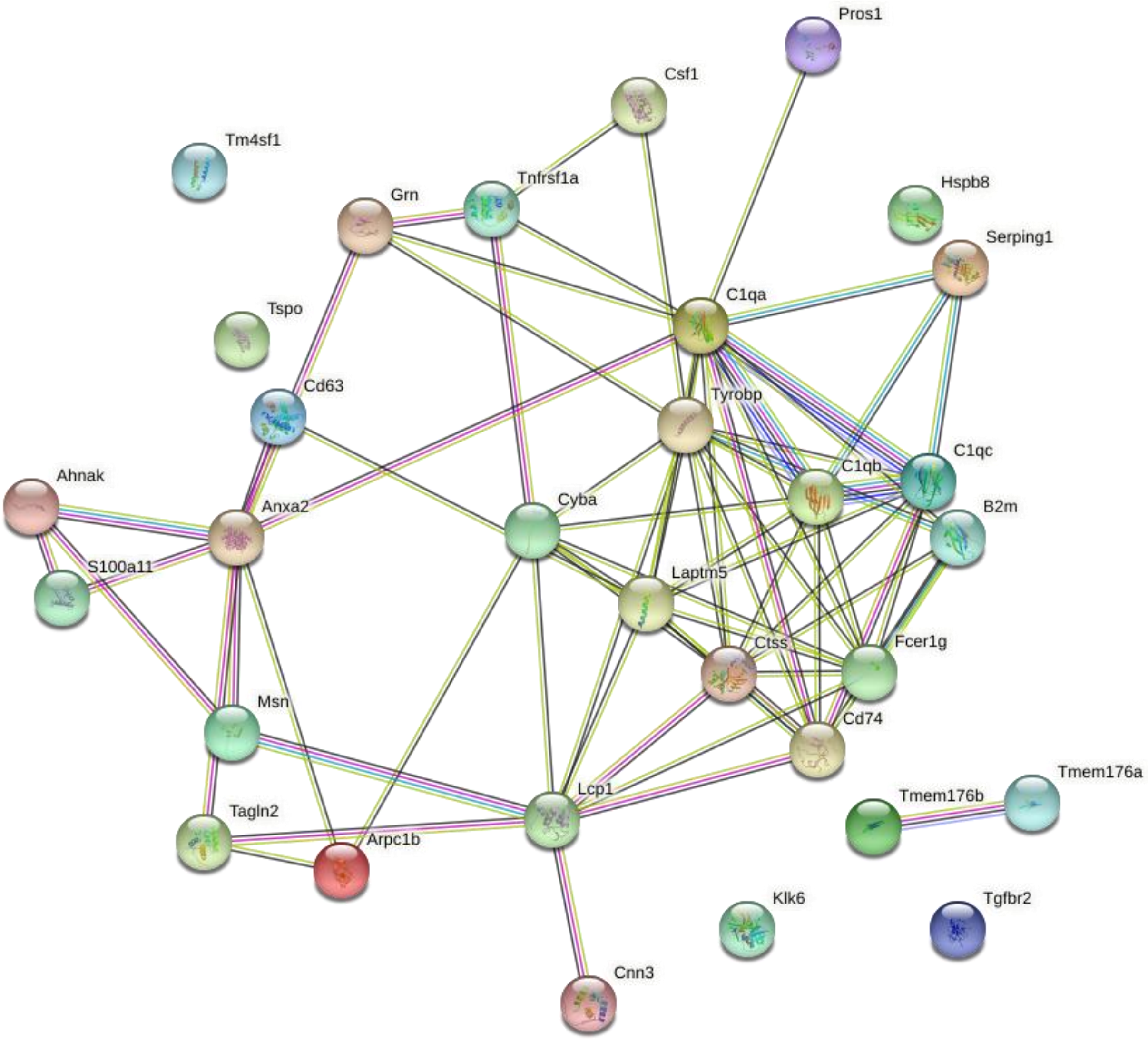

Number of nodes: 31
Number of edges: 72
Average node degree: 4.65
Avg. local clustering coefficient: 0.511
Expected number of edges: 7
Protein-protein interaction (PPI) enrichment p-value < 1.0e-16

Figure S5: TgTau^{P301L} with frontal pathology vs negative pathology

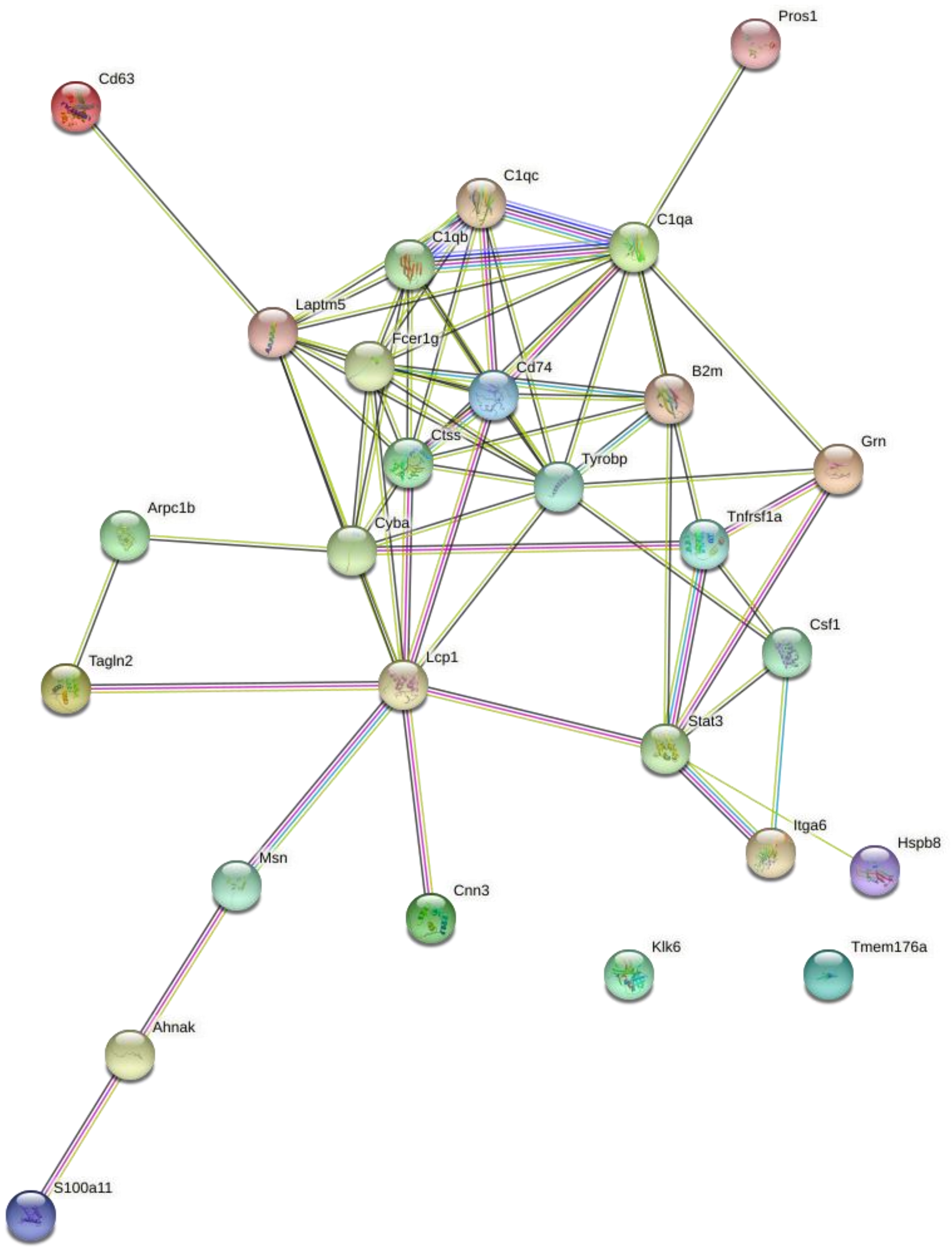

Number of nodes: 27
Number of edges: 68
Average node degree: 5.04
Avg. local clustering coefficient: 0.543
Expected number of edges: 8
Protein-protein interaction (PPI) enrichment p-value: < 1.0e-16

Figure S6

A

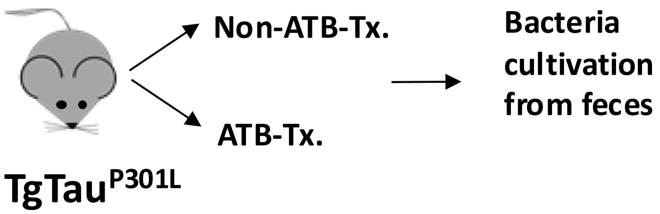
Non-ATB-Tx.
ATB-Tx.
Bacteria cultivation from feces
TgTau P301L

B

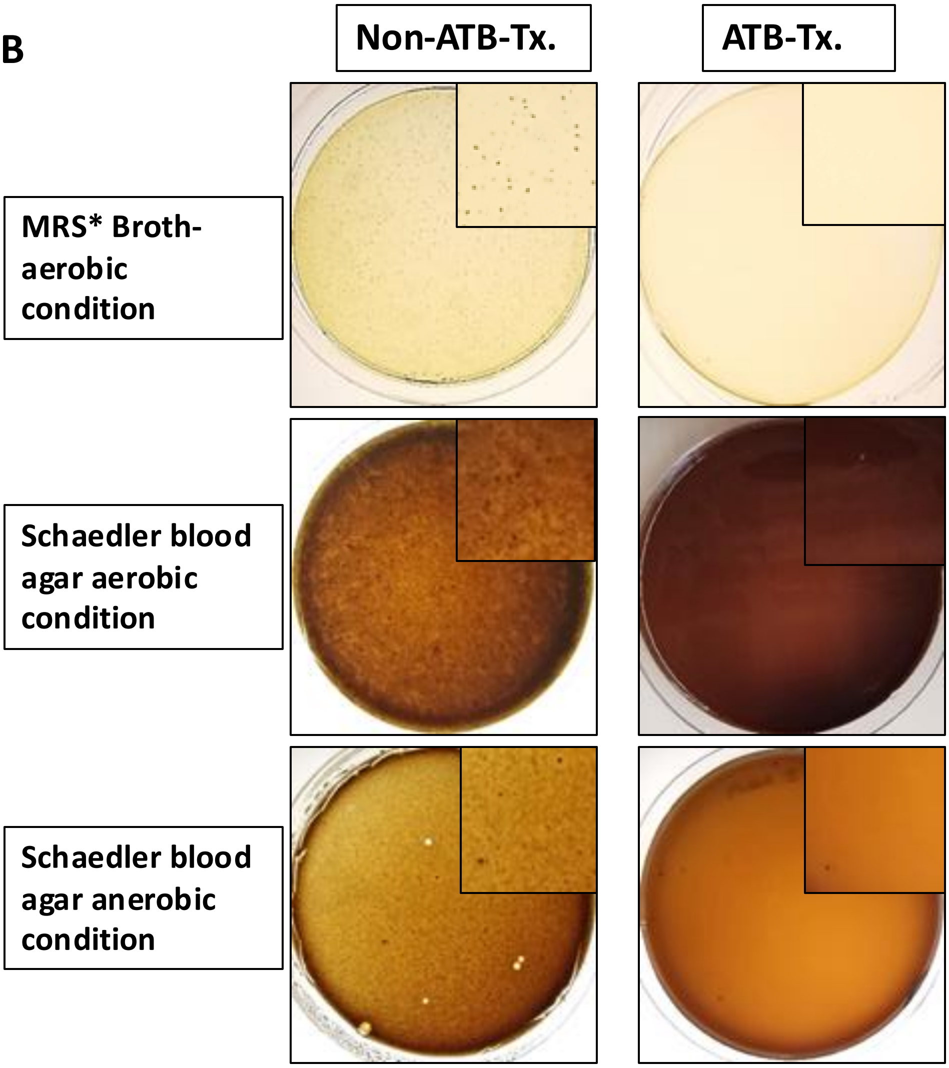
Non-ATB-Tx.
ATB-Tx.
MRS* Broth-aerobic condition
Schaedler blood agar aerobic condition
Schaedler blood agar anerobic condition

Supplementary Tables

Diversity / Variable	Shannon index				Simpson index				Observed index
Taxonomic level	Family	Genus	Species	OTU	Family	Genus	Species	OTU	Phylum, Class, Order, Family, Genus and Species
[Kruskal-Wallis] statistic	8,8165	10.027	9.9542	9.9542	13	13.205	13.173	13.173	ns
p-value	0.0318	0.0183	0.0190	0.0190	0.0052	0.0042	0.0043	0.0043	ns

Table S1. Alpha diversity analysis at different taxonomic levels for 12Svev/Tac and C57BL/Tac mice samples

Diversity / Variable	Shannon index		Simpson index			Observed index
Taxonomic level	Class	Order	Phylum	Class	Order	Phylum, Class, Order, Family, Genus and Species
[Kruskal-Wallis] statistic	9,532	11.281	11.188	11.209	10.688	n.s.
p-value	0.0230	0.0103	0.0108	0.0107	0.0135	n.s

Table S2. Alpha diversity analysis at different taxonomic levels for C57BL/Tac mice samples.

Taxa	Phylum	Class	Order	Family	Genus	Species
OTU0111	TM7-3o	CW040f	F16g			
OTU0041	Bacillio	Lactobacillalesf	Lactobacillaceaeg	Lactobacillus		
OTU0017	Bacteroidiao	Bacteroidalesf	Prevotellaceaeg	Prevotellas		
OTU0026	Bacteroidiao	Bacteroidalesf	S24-7g			
OTU0063	Clostridiao	Clostridialesf	Lachnospiraceaeg	Lachnospiras		
OTU0103	Epsilonproteobacteriao	Campylobacteralesf	Helicobacteraceaeg	Flexispira		
OTU0069	Clostridiao	Clostridialesf	Lachnospiraceaeg	Roseburias	inulinivorans	
OTU0104	Epsilonproteobacteriao	Campylobacteralesf	Helicobacteraceaeg	Helicobacter		

Table S3. Taxonomic description of selected OTU from Figure 3C.

Supplementary Tables

Diversity Variable	Shannon index	Simpson index		Observed index
**Taxonomic level**	Phylum, Class, Order, Family, Genus and Species	Species	OTU	Phylum, Class, Order, Family, Genus and Species
**[Kruskal-Wallis] statistic**	n.s.	**7.757**	**7.757**	n.s.
**p-value**	n.s	0.0513	0.0513	n.s

Table S4. Alpha diversity analysis at different taxonomic levels for samples of 129SvEv /Tac mice.

Taxa	Phylum	Class	Order	Family	Genus	Species
**OTU0079**	Clostridiao	Clostridialesf	Ruminococcaceaeg	Oscillospiras		
**OTU0080**	Clostridiao	Clostridialesf	Ruminococcaceaeg	Oscillospiras	guilliermondii	
**OTU0060**	Clostridiao	Clostridialesf	Lachnospiraceaeg	Clostridium		
**OTU0082**	Clostridiao	Clostridialesf	Ruminococcaceaeg	Ruminococcuss		
**OTU0091**	Alphaproteobacteriao	RF32f				

Table S5. Taxonomic description of selected OTU from Figure 3 down.
